# Supplementary material for: Multi‐dataset identification of innovative feature genes and molecular mechanisms in keratoconus
Source: J Cell Mol Med. 2024 Sep 19;28(18):e70079. doi: 10.1111/jcmm.70079 (PMC11412914; doi:10.1111/jcmm.70079)
Supplement: Supplementary file 2 — Material S2. [file JCMM-28-e70079-s002.zip › 7.GSEA analysis/5.RNF217-AS1/KEGG_PENTOSE_PHOSPHATE_PATHWAY.html]

Details for gene set KEGG\_PENTOSE\_PHOSPHATE\_PATHWAY[GSEA]

|  || Dataset | RNF217-AS1.group.cls#h\_versus\_l |
| Phenotype | group.cls#h\_versus\_l |
| Upregulated in class | h |
| GeneSet | KEGG\_PENTOSE\_PHOSPHATE\_PATHWAY |
| Enrichment Score (ES) | 0.22003403 |
| Normalized Enrichment Score (NES) | 0.83831465 |
| Nominal p-value | 0.74747473 |
| FDR q-value | 1.0 |
| FWER p-Value | 1.0 |
Table: GSEA Results Summary

  

Fig 1: Enrichment plot: KEGG\_PENTOSE\_PHOSPHATE\_PATHWAY      
 Profile of the Running ES Score & Positions of GeneSet Members on the Rank Ordered List

  

| SYMBOL | TITLE | RANK IN GENE LIST | RANK METRIC SCORE | RUNNING ES | CORE ENRICHMENT || 1 | TKTL1 | na | 280 | 0.606 | 0.2200 | Yes |
| 2 | PFKP | na | 8076 | 0.126 | 0.0428 | No |
| 3 | ALDOC | na | 8349 | 0.120 | 0.0800 | No |
| 4 | TKTL2 | na | 8415 | 0.118 | 0.1227 | No |
| 5 | RPEL1 | na | 10697 | 0.075 | 0.0851 | No |
| 6 | FBP1 | na | 12030 | 0.055 | 0.0674 | No |
| 7 | RBKS | na | 12802 | 0.045 | 0.0620 | No |
| 8 | RPE | na | 13464 | 0.037 | 0.0568 | No |
| 9 | TALDO1 | na | 16666 | 0.002 | -0.0347 | No |
| 10 | PGLS | na | 17060 | -0.002 | -0.0451 | No |
| 11 | ALDOA | na | 17684 | -0.008 | -0.0601 | No |
| 12 | TKT | na | 18938 | -0.019 | -0.0889 | No |
| 13 | PGM2 | na | 19090 | -0.021 | -0.0854 | No |
| 14 | GPI | na | 19976 | -0.030 | -0.0997 | No |
| 15 | RPIA | na | 20691 | -0.037 | -0.1064 | No |
| 16 | PFKL | na | 20751 | -0.037 | -0.0941 | No |
| 17 | G6PD | na | 21848 | -0.049 | -0.1072 | No |
| 18 | DERA | na | 22660 | -0.058 | -0.1088 | No |
| 19 | PRPS1 | na | 23203 | -0.064 | -0.1003 | No |
| 20 | PFKM | na | 23341 | -0.066 | -0.0794 | No |
| 21 | PGM1 | na | 25371 | -0.096 | -0.1015 | No |
| 22 | PGD | na | 25517 | -0.099 | -0.0685 | No |
| 23 | PRPS2 | na | 26541 | -0.117 | -0.0540 | No |
| 24 | FBP2 | na | 28391 | -0.158 | -0.0476 | No |
| 25 | H6PD | na | 29481 | -0.191 | -0.0073 | No |
| 26 | PRPS1L1 | na | 29738 | -0.198 | 0.0599 | No |
| 27 | ALDOB | na | 30478 | -0.223 | 0.1225 | No |
Table: GSEA details [plain text format]

  

Fig 2: KEGG\_PENTOSE\_PHOSPHATE\_PATHWAY      
 Blue-Pink O' Gram in the Space of the Analyzed GeneSet

  

Fig 3: KEGG\_PENTOSE\_PHOSPHATE\_PATHWAY: Random ES distribution      
 Gene set null distribution of ES for **KEGG\_PENTOSE\_PHOSPHATE\_PATHWAY**
